# Evaluation of Sentinel-2 Red-Edge Bands for Empirical Estimation of Green LAI and Chlorophyll Content

**DOI:** 10.3390/s110707063

**Published:** 2011-07-08

**Authors:** Jesús Delegido, Jochem Verrelst, Luis Alonso, José Moreno

**Affiliations:** Department of Earth Physics and Thermodynamics, Image Processing Laboratory, Universidad de Valencia, P.O. Box 22085, Paterna E-46071,Valencia, Spain; E-Mails: jochem.verrelst@uv.es (J.V.); luis.alonso@uv.es (L.A.); jose.moreno@uv.es (J.M.)

**Keywords:** Sentinel-2, chlorophyll, LAI, NAOC, NDI, red-edge

## Abstract

ESA’s upcoming satellite Sentinel-2 will provide Earth images of high spatial, spectral and temporal resolution and aims to ensure continuity for Landsat and SPOT observations. In comparison to the latter sensors, Sentinel-2 incorporates three new spectral bands in the red-edge region, which are centered at 705, 740 and 783 nm. This study addresses the importance of these new bands for the retrieval and monitoring of two important biophysical parameters: green leaf area index (LAI) and chlorophyll content (Ch). With data from several ESA field campaigns over agricultural sites (SPARC, AgriSAR, CEFLES2) we have evaluated the efficacy of two empirical methods that specifically make use of the new Sentinel-2 bands. First, it was shown that LAI can be derived from a generic normalized difference index (NDI) using hyperspectral data, with 674 nm with 712 nm as best performing bands. These bands are positioned closely to the Sentinel-2 B4 (665 nm) and the new red-edge B5 (705 nm) band. The method has been applied to simulated Sentinel-2 data. The resulting green LAI map was validated against field data of various crop types, thereby spanning a LAI between 0 and 6, and yielded a RMSE of 0.6. Second, the recently developed “Normalized Area Over reflectance Curve” (NAOC), an index that derives Ch from hyperspectral data, was studied on its compatibility with simulated Sentinel-2 data. This index integrates the reflectance curve between 643 and 795 nm, thereby including the new Sentinel-2 bands in the red-edge region. We found that these new bands significantly improve the accuracy of Ch estimation. Both methods emphasize the importance of red-edge bands for operational estimation of biophysical parameters from Sentinel-2.

## 1. Introduction

Global Monitoring for Environment and Security (GMES) is a joint initiative of the European Commission and the European Space Agency (ESA), designed to establish a European capacity for the provision and use of operational monitoring information for environment and security applications [1]. Given the fact that the current services are based on data from Landsat and SPOT sensors, a satisfactory service could be expected by continuing these mission programmes as a minimum scenario. However, with a view to demanded service improvements in the near future, an enhanced land surface monitoring system in terms of spectral, temporal and spatial coverage is required. The upcoming Sentinel-2 (S2) mission intends to provide such continuity to services, but with improved features compared to the later sensors [1].

S2 is a polar-orbiting, superspectral high-resolution imaging mission designed for GMES land monitoring. The mission is envisaged to fly a pair of satellites, with the first planned to be launched in 2013. Each S2 satellite carries a Multi-Spectral Imager (MSI) with a swath of 290 km. It provides a versatile set of 13 spectral bands spanning from the visible and near infrared to the shortwave infrared, featuring four bands at 10 m, six bands at 20 m and three bands at 60 m spatial resolution [2]. Furthermore, S2 incorporates three new bands in the red-edge region, which are centered at 705, 740 and 783 nm. In full operational phase, the pair of S2 satellites will deliver data taken over all land surfaces and coastal zones every five days under cloud-free conditions, and typically every 15–30 days considering the presence of clouds. To serve the objectives of GMES, S2 satellites will provide imagery for the generation of high-level operational products (Level 2b/3) such as land-cover maps, land-change detection maps, and plants geophysical variables, such as chlorophyll content per unit leaf area (Ch), leaf area index (LAI) and leaf water content [2].

Effectively, spatially-explicit knowledge of vegetation’s Ch and LAI is fundamental for the understanding of agricultural and forested ecosystems [3,4]. Ch can be considered as a bio-indicator of plants’ actual health status [5,6], and of vegetation gross primary productivity [7]. Further it is one of the main inputs in plant growth models. Also green LAI, defined as the total of one-sided area of green leaves per ground area [8] represent a key parameter, characterizing the structure and functioning of vegetation cover [9]. Due to its role as the interface between ecosystem and atmosphere and involvement in many processes, green LAI is a key variable in aboveground biomass estimation, vegetative evapotranspiration calculation and the energy-exchange evaluation of terrestrial vegetation [10–15].

Fundamentally, the retrieval of a biophysical variable such as Ch and LAI from earth observation data always implies the use of a model [16]. This model can be either empirical or physical. Empirical models directly link Earth observation (EO) data with the variables of interest, e.g., through statistical approaches. Physical models refer to the inversion of radiative transfer models (RTM) against EO data to obtain the variables of interest [17–20]. Concerning physical models, experimental studies using RTMs have shown great flexibility in retrieving plant cover variables, because of being able to parameterize these models to a wide range of land cover situations and sensor configurations [21,22]. However, two main drawbacks limit the use of RTMs for operational applications. First, RTM approaches typically require auxiliary information per land cover type to parameterize the model, which may not always be available [23]. An additional problem hereby is that if uncertainties are introduced the likelihood increases that the model inversion will not lead to a unique solution (unified theorem of Hadamard well-posedness) and extra steps are required to overcome the ill-posed problem [24]. Second, regardless of the availability of auxiliary data, for the majority of the RTMs that are fast enough for operational applications there is the intrinsic risk of oversimplifying the architecture of plant cover [25,26]. For instance, a recent study concluded that a RTM approach using the SAIL model was unable to cope with the strong leaf clumping in row crops such as maize for simulated S2 data [27].

Alternatively, empirical models are more straightforward implementable in an operational data processing chain. Over the past four decades, a large number of spectral indices (e.g., vegetation indices) have been developed for the study of biophysical variables such as LAI or Ch [16,28–31]. While these spectral indicators do not rely on auxiliary data, their limitations rather lie in the nature of its empirical design. Empirical methods tend to impose uncertainties when applied to conditions other than those wherein the model was initially developed, such as other atmospheric conditions, sensor configurations, sun-target-sensor geometry, or when upscaled from leaf-to-canopy [32,33]. Conversely, with the advent of hyperspectral imaging, many novel algorithms have been developed over the last few years, which have shown to be more accurate and robust in estimating canopy parameters than traditional spectral indices. These novel algorithms typically make use of more or better band combinations on the one hand, or of a continuous spectral range on the other [34,35].

When it comes to the implementation of a candidate retrieval method into a S2 data processing chain, nevertheless, crucial is to invest in prediction models that are simple, robust and generally applicable. This implies that the dependency on ancillary data should be kept to the minimum. Novel empirical algorithms may therefore be preferred above physical models in an operational context, given that their robustness across various crop types is sufficiently tested. In this respect, we propose two simple yet accurate empirical algorithms that derive green LAI and Ch from simulated S2 data. Specifically, in this work it was of interest to test the efficacy of the proposed methods across various crop types, and to evaluate the importance of the new red-edge bands when applying these methods to S2 data. Given all the above, the objective of the present study was twofold: (*i*) to evaluate the capability of two novel empirical models assessing green LAI and Ch from simulated S2 data, and thereby (*ii*) to evaluate the added value of the spectral bands in the red-edge region.

## 2. Methods

### 2.1. Spectral Indices

Vegetation indices are among the oldest and most widely used tools to estimate Ch and LAI (e.g., see reviews in Bannari *et al.* [36], He *et al.* [37], Haboudane *et al.* [38], Zheng and Moskal [21]). Vegetation indices are simple numerical indicators that reduce multispectral (two or more spectral bands) data to a single variable for predicting and assessing vegetation characteristics. The best understood index is probably the Normalized Difference Vegetation Index (NDVI), originally proposed by Rouse *et al.* [39] as:

(1) $$\text{NDVI}=\frac{{\text{R}}_{800}-{\text{R}}_{670}}{{\text{R}}_{800}+{\text{R}}_{670}}$$

where R_i_ is reflectance at the band centered at a given wavelength *i* (in nm). This index has been applied in numerous studies on, amongst others, plant development, Ch, green biomass, nitrogen content and LAI [40–42]. Apart from the NDVI, numerous alternative indices have been proposed showing sensitivity towards LAI [38,41,43–46]. Many of these indices use bands in the red-edge region [45,47–49]. For instance, Gitelson and Merzlyak [48] proposed an NDVI-like index using 705 and 750 nm bands for assessing Ch. At the same time, while having more and more spectral bands available, eventually all two-band combinations can be calculated in the form of a generic Normalized Difference Index (NDI), defined as:

(2) $$\text{NDI}=\frac{{\text{R}}_{\text{b}}-{\text{R}}_{\text{a}}}{{\text{R}}_{\text{b}}+{\text{R}}_{\text{a}}}$$

and looking for those wavelengths *a* and *b* that provide the best correlation with LAI, Ch and some other biophysical parameters obtained from experimental data [34,42,50,51]. Specifically, Zhao *et al.* [50] demonstrated that for low LAI values an optimized NDI can be related with LAI by means of a linear regression. Although the index tends to become saturated for an LAI above 5 [52,53], commonly LAI of crops remain below this saturation threshold. Hence, this suggests that a well-chosen NDI would be a simple and successful method to predict LAI of crops. It is therefore of interest to find the optimal couple of bands for NDI that provide the maximum linear correlation with LAI given data from agricultural areas as has been demonstrated in associated work [54] where, by using hyperspectral CHRIS (Compact High Resolution Imaging Spectroscopy) data, it was found that LAI can be best estimated with bands centered at 712 and 674 nm for R_b_ and R_a_, respectively. From 300 measurements obtained across seven different crop types and bare soils, with values of LAI between 0 and 7, it led to the following linear relationship [54]:

(3) $$\text{LAI}=6.753\left(\frac{{\text{R}}_{712}-{\text{R}}_{674}}{{\text{R}}_{712}+{\text{R}}_{674}}\right)\;\;\text{r}=0.908$$

where r is the correlation coefficient.

On the other hand, when having many (narrow) bands available it is also possible to derive vegetation characteristics using a more continuous approach instead of using only two bands. In this respect, recently the Normalized Area Over the reflectance Curve (NAOC) index was developed to estimate Ch, and is defined as [35]:

(4) $$\text{NAOC}=1-\frac{\int_{\text{a}}^{\text{b}}\text{R}\cdot \text{d}\lambda }{{\text{R}}_{\text{b}}(\text{b}-\text{a})}$$

where λ is the wavelength, R_b_ is the maximum far-red reflectance, corresponding to reflectance at the wavelength *b*, and *a* and *b* are the integration limits surrounding the Ch absorption centered at ∼670 nm. In [35], best results from NAOC in estimating Ch were obtained with the integration limits from *a* = 643 nm to *b* = 795 nm, resulting in a final expression for NAOC given by:

(5) $$\text{NAOC}=1-\frac{\int_{643}^{795}\text{R d}\lambda }{152\;\;{\text{R}}_{795}}$$

In the same study, the relationship between NAOC and Ch was obtained:

(6) Ch=−3.8868+101.94 NAOC                r=0.909

where Ch is in μg/cm^2^ [35].

NAOC proved to act as a reliable predictor of Ch; a recent study comparing the predictive power of NAOC against 32 established indices sensitive to Ch found that NAOC obtained an accuracy that ended in the top three [55].

This paper focussed on evaluating the compatibility of the aforementioned empirical methods with the envisaged S2 band configuration (see Table 1). Other characteristics of the S2 instrument such as spatial size and signal-to-noise have not been considered in the analysis. S2 band configuration provides three spectral bands in the red-edge region: bands B5 and B6 located at the sharp edge, and B7 that is located at the shoulder of the NIR plateau. These three bands and the B4 band lie right within the NAOC integration limits.

### 2.2. Experimental Data

We used data from three recent ESA field campaigns: SPARC, AgriSAR and CEFLES2. Each of these campaigns was dedicated to an improved understanding of the interactions between solar radiation, plant cover and atmosphere through the use of novel EO instruments. During these campaigns images were acquired from various airborne and spaceborne sensors and a multitude of vegetation structural, functional and radiometric characteristics were measured. The purpose of the campaigns is briefly explained below, information about crop types, field measurements, sensors and preprocessing steps is listed in Table 2.

- The 2003 and 2004 Spectra Barrax Campaigns (SPARC) took place at Barrax (La Mancha region, Spain). These campaigns aimed to collect coincident field data over the Barrax site with CHRIS multi-angular and hyperspectral. An extensive data set was collected, covering soil, vegetation and atmospheric parameters. A large set of ground sampling points were identified. Each ground point is called elementary sample units (ESU). LAI and Ch were measured in a circle of radius 10 m with a size equivalent to a CHRIS pixel. LAI was derived from canopy measurements made with a LiCor LAI-2000 digital analyzer [57].
- The AgriSAR (Airborne SAR and Optics) campaign ran for four months, from the 18 April to 2 August 2006 in Demmin (Germany), with a data collection of approximately every week. The AgriSAR project aimed to build a database for the investigation and validation of the retrieval of biophysical parameters and simulating Sentinel-1 and -2 image products over the land. In each ESU, LAI was derived from canopy measurements made with a LiCor LAI-2000 [58,59].
- CEFLES2 (CarboEurope, FLEX and S2) was located in the Les Landes region, southwest of France. During three measurement periods in April, June and September 2007 focus was on various landscape types, including urban, agricultural and forested areas. These periods span the beginning and peak of the vegetation growing cycle and post-harvest in order to broaden the availability of data from different crops and phenological states [60]. Chlorophyll a + b were measured with a calibrated [58] field chlorophyll meter (SPAD-502). The methodology applied to obtain *in situ* Ch data at each ESU consisted of measuring about 50 samples with the SPAD and then calculating its average.

### 2.3. LAI and Ch Estimation from S2 Data

The first focus was on the estimation of LAI. Data from the SPARC campaign was used as a reference dataset because of spanning a wide variety of crop types and LAI values [57,61]. Four hyperspectral CHRIS acquisition sets were obtained in the 2003 and 2004 campaigns; from them only the ones corresponding to nadir view were selected so that angular and atmospheric effects are minimized and that highest spatial resolution is preserved. 240 elementary sample units (ESUs) plots from crops and additional 60 samples from bare soils and the corresponding spectra were extracted. From the acquired CHRIS spectra, NDI values were calculated according to Equation 3 and plotted against the corresponding reference LAI values. Of specific interest here is to evaluate its compatibility with the S2 band settings. CHRIS is well suited for assessing the performance of the upcoming S2 sensor given its spectral similarity in the visible and NIR. The sensor overlaps the S2 bands up to B9 (945 nm), although there is some difference in bandwidth: CHRIS bandwidth ranges between 1.3 nm and 11.3 nm while S2 bandwidth ranges between 15 nm and 180. Two S2 bands approach closely to the CHRIS bands centered at 674 and 712 nm, being B4 and B5 (Table 2). B4 centered at 665 nm coincident with chlorophyll’s maximum absorption, and B5 centered at 705 nm in the red-edge region. B5 is one of the new bands incorporated in this mission aiming to improve vegetation monitoring [56]. In turn, when comparing the S2 band settings with those of CHRIS, two CHRIS bands in mode 1 are positioned approximately within the centre of those S2 bands, with similar yet slightly smaller bandwidth. It is therefore worthwhile to apply these bands as a substitute of S2 bands in an NDI and correlate again with LAI measurements.

The second focus was on the estimation of canopy chlorophyll, which in this work was derived from the NAOC index (Equation 5). NAOC has been earlier used with CHRIS data from the SPARC data set [35]. Here, the emphasis lied on assessing the robustness of the NAOC and its compatibility with S2 band settings. Leaf Ch and LAI measurements from the CEFLES2 project during the September 2007 campaign were used to establish a relationship between NAOC and canopy chlorophyll. Field data included four crop types: corn, bean, sunflower and kiwi trees. NAOC was calculated from an atmospherically corrected AHS (Airborne Hyperspectral System) image acquired over the Marmande test site (Landes region). Subsequently, the AHS imagery has been resampled to the S2 band settings. NAOC was again calculated in two different ways: with red-edge bands included (*i.e*., using B4, B5, B6 and B7) and without red-edge bands (*i.e*., using B4 and B7 bands only). Finally, both NAOC maps have been compared on their performances in canopy chlorophyll estimation.

## 3. Results

### 3.1. LAI Estimation

**Figure 1.**
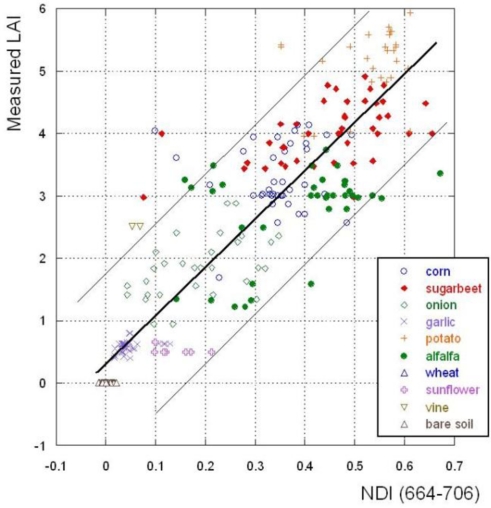
Measured LAI against NDI from 664 and 706 nm from CHRIS data. Central line corresponds to Equation 7 and the finest lines plus and minus twice the standard deviation.

LAI measurements from the SPARC campaign were plotted against the NDI calculated with the S2-like selected CHRIS bands centered at 664 and 706 nm in Figure 1. The resulting scatter plot was fitted to the following linear equation:

(7) $$\text{LAI}=8.452\left(\frac{{\text{R}}_{706}-{\text{R}}_{664}}{{\text{R}}_{706}+{\text{R}}_{664}}\right)\;\;\;\text{r}=0.903$$

which can be used to estimate green LAI from S2 bands B4 and B5. Because a wide range of crop types were included, this equation is applicable for estimating green LAI over multiple agricultural sites. Unfortunately no field data with LAI above 6 was available, leaving uncertainty about the validity of the relationship at high LAIs. Although a few crop types with appreciable leaf production may exceed this value for a short time period, such as corn prior to senescing, in fact the majority of crops types stay well below this value during the entire growing cycle [63], making this equation of interest for further evaluation.

To validate the utility of the proposed equation for LAI retrievals from future S2 images, field data and spectral observations from a different campaign, the AgriSAR campaign, was used. During AgriSAR, airborne hyperspectral CASI (Compact Airborne Spectrographic Imager) images were acquired over agricultural areas. From the different flightlines available, the images that cover most ground sampling points were selected.

**Figure 2.**
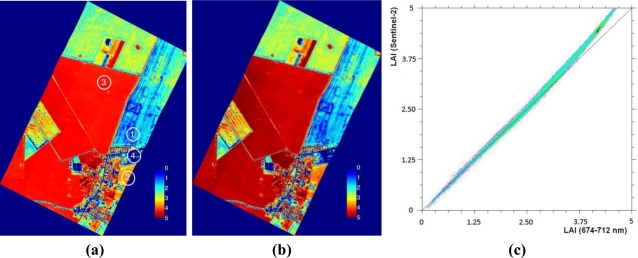
**(a)** Green LAI map derived from CASI data using NDI on bands at 674 and 712 nm; **(b)** Green LAI map from S2 bands B4 and B5. Numbers on the 2a map indicate the locations used for validation; **(c)** Scatter plot of the LAI maps derived from CASI and S2 data using NDI.

A LAI map was produced by applying Equation 3 to the CASI image, shown in Figure 2(a). A similar map was calculated using Equation (7) with spectrally resampled data according the band settings of S2. This map is displayed in Figure 2(b). Comparing Figure 2(a,b), it can be observed that both maps provide very similar results. This is also apparent when comparing both maps in a scatter plot [Figure 2(c)]. Both maps consistently follow the one-to-one line until a LAI of about 4 is reached, then the S2-based map start to slightly overestimate the higher LAI values, though the values never exceeded a deviation of 0.4. The overall good relationship illustrates that the above-described method can be easily applied to S2 data.

**Figure 3.**
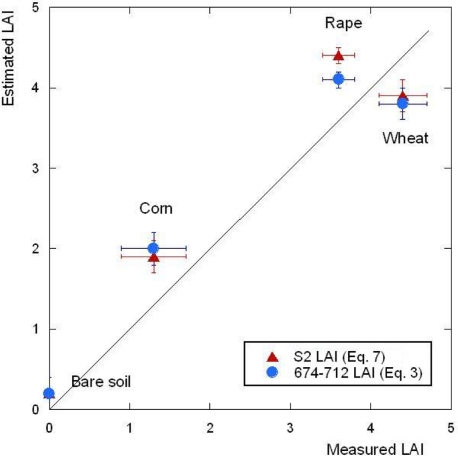
Scatter plot of *in situ* measured *versus* estimated green LAI values according to Equation 3 and Equation 7 from AgriSAR data with corresponding error bars.

The proposed Equations (3) and (7) were validated using *in-situ* LAI measurements of the AgriSAR campaign on 4 July 2006. The ground area of Figure 2 covered three crop types: corn, wheat and rape. Six field measurements were collected and averaged for each crop type and also bare soil surface measurements were included (0 LAI). The measured values were compared with corresponding estimated LAI as extracted from the CASI-based and S2-based LAI maps. It led to a root mean square error (RMSE) of 0.53 and 0.57, respectively. Figure 3 depicts the correlation between *in situ* measured and calculated LAI values, showing a good agreement in magnitude, given the small number of samples per crop type. There were only marginal differences in the performance between the CASI-based NDI (Equation (3)) and the S2-based NDI (Equation (7)). The proposed NDI formulation with S2 bands B4 and B5 can therefore be considered as a useful estimator of green LAI from S2 data.

### 3.2. Canopy Chlorophyll Estimation

#### 3.2.1. Calibration

**Figure 4.**
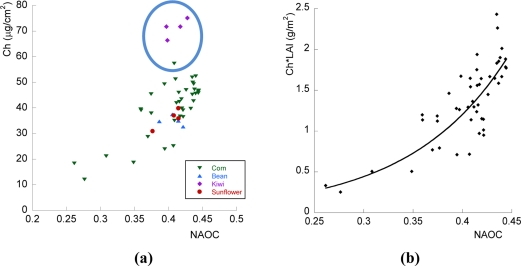
**(a)** Ch as a function of AHS derived NAOC. Some points that fall outside the general trend correspond to kiwi, with high Ch but low LAI; **(b)** Correlation of NAOC with leaf chlorophyll multiplied by LAI. Resulting canopy chlorophyll is expressed as gram chlorophyll per square soil meter.

The second focus of this study involved the estimation of canopy chlorophyll, and the role that red-edge bands can play herein. Measurements from the CEFLES2 campaign were plotted against corresponding values from the AHS-based calculated NAOC map [Figure 4(a)]. It can be observed that the relationship between Ch and NAOC agrees, even along various crop types. However, some measurements with highest Ch values seem to deviate from the general trend. They correspond to kiwi plants, which have very high chlorophyll at leaf level, but on the other hand, kiwi trees present a relatively thin crown, and the stands are several meters apart from each other. When viewed from an air- or space-borne platform, this consequently results in a low density of leaves per pixel. To correct for this, it is necessary to relate the canopy level Ch-index to canopy level chlorophyll instead of Ch.

Although Ch distribution in the plant is not necessarily uniform, taking into account that LAI represents the portion of green leaves per ground area, the product chlorophyll by LAI (Ch*LAI) provides an indication of the total chlorophyll content per unit ground area in the canopy [64,65]. This product was plotted against NAOC in Figure 4(b) and the resulting distribution has been fitted to an exponential equation:

(8) $$\text{Ch}*\text{LAI}=0.0219\;{\text{e}}^{10.02\;\text{NAOC}}\;\;\;\;\text{r}=0.795$$

which serves as calibration for the index. With this exponential equation it is possible to derive a canopy chlorophyll map from the NAOC map. In the following section the performance of the equation is evaluated given spectrally resampled data according to the band configuration of S2.

#### 3.2.2. Application to Simulated S2 Data & Effect of Red-Edge Bands in Chlorophyll Map

**Figure 5.**
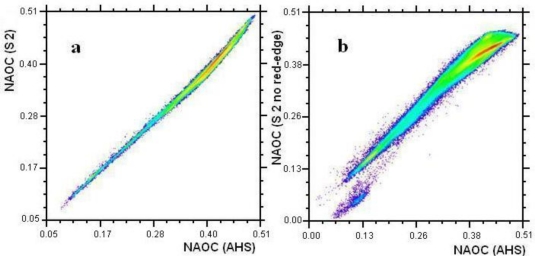

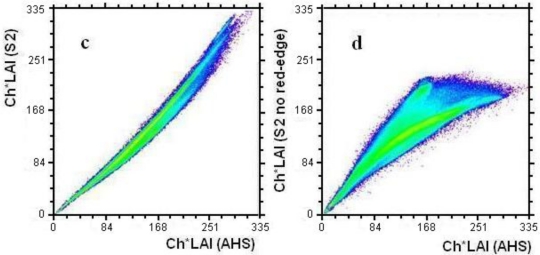
Scatter plots. **(a)** S2-based NAOC against AHS-based NAOC; **(b)** S2-based NAOC calculated without red-edge bands against AHS-based NAOC; **(c)** S2-based Ch*LAI against AHS-based Ch*LAI; and **(d)** S2-based Ch*LAI calculated without red-edge bands against AHS-based Ch*LAI. The colour scale indicates pixel density.

The NAOC index was used to evaluate the importance of the S2 red-edge bands in assessing canopy chlorophyll. The S2 bands needed for calculating NAOC are B4 to B7, with B5 and B6 as red-edge bands. NAOC was first calculated from the AHS image (63 bands between 430 and 2500 nm) using all bands between 643 and 795 nm, which served as reference map. The AHS image was then spectrally resampled to two new images with the band settings of S2, but the second one without red-edge bands. Scatter plots of both S2-based NAOC maps (with red-edge bands, *i.e*., using B4, B5, B6 and B7 bands, and without red-edge bands *i.e*., using B4 and B7 bands only) against the reference AHS-based NAOC map show the degree of correlations. The S2-based NAOC map correlated closely with the AHS-based NAOC map [Figure 5(a)], indicating that the coarser spectral sampling of S2 does not substantially downgrade the results. Conversely, the S2-based NAOC map without the red-edge bands led to considerably poorer correlations [Figure 5(b)], especially at higher values, which are the ones related to more dense vegetation (green vegetation corresponds to NAOC values larger than 0.35). Canopy chlorophyll maps were subsequently derived from the NAOC data by using the above-proposed exponential relationship (Equation 8), and scatter plots were again created against the AHS reference data (Figure 5). Note that despite small effects of underestimation due to the coarser spectral sampling the S2 map obtained with red-edge bands holds a strong correlation [Figure 5(c)]. At the same time, in absence of red-edge bands, the exponential relationship between NAOC and canopy chlorophyll amplified the slight misfit in NAOC [Figure 5(b)], until a point where correlation is lost and saturation starts to appear [Figure 5(d)]. Hence, as the absence of red-edge bands in the proposed algorithm lead to systematic erroneous retrievals, these scatter plots underpin the relevance of these bands.

**Figure 6.**
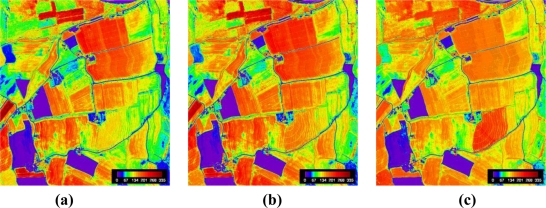
Canopy chlorophyll (Ch*LAI) maps, derived from: **(a)** simulated S2 data; **(b)** AHS data; and **(c)** simulated S2 data without red-edge bands.

Finally, canopy chlorophyll maps were obtained by applying the calibration function (Equation (8)) to the NAOC maps. The results displayed in Figure 6 show that estimated chlorophyll from S2 image [Figure 6(a)] is practically the same than the one estimated from the original AHS image [Figure 6(b)]. In turn, the chlorophyll map derived from S2 without the red-edge bands shows clear differences. Discrepancies in the absence of red-edge bands are to be found over the various maize fields throughout the map [e.g., compare Figure 6(b) with Figure 6(c)].

## 4. Discussion

To fulfill the monitoring needs of the GMES land services and research communities for years to come S2 aims to ensure continuity on the technology and the experience acquired by the SPOT and Landsat families, and to deliver improved operational high-level products [56]. These goals ultimately led to the design of a multi-spectral imager (MSI) that is not only configured with the same spectral bands as the latter sensors, but also incorporates two new bands that exploit the red-edge information. At the same time, this improved sensor configuration pursued the need for improved biophysical parameter retrieval algorithms [2]. In this work we assessed the importance of the S2 red-edge bands with respect to the retrieval of green LAI and canopy chlorophyll content. Therefore, two algorithms that specifically make use of new bands in red-edge, being an optimized NDI and NAOC, were evaluated on its compatibility using spectrally resampled data given the proposed S2 band configuration. While the importance of red-edge bands has been addressed in earlier studies [49,66], in this work we found that the inclusion of these bands are important for S2 to enable the delivery of accurate green LAI and canopy chlorophyll products. NDI led to best correlations with green LAI through the use of a red-edge band B4 [54], and also NAOC needed the red-edge bands (B4 and B5) to achieve precise correlations with canopy chlorophyll. Through band-specific efficiency analysis techniques (e.g., [67]), the importance of the red-edge region in two forthcoming superspectral sensors (S2 and VENμS) was also stressed by Hermann *et al.* [68]. These results are encouraging for the upcoming S2 mission. We are assured that the inclusion of red-edge bands will advance the quality of high level products.

Emphasis was put on validating the performance of the methods with data from various test sites. Regarding the LAI-optimized NDI, data from two ESA-led field campaigns were used: SPARC and AgriSAR. Validation over various crop types yielded satisfactory results; the S2 band setting led to a RMSE of 0.6. This is encouraging, taking into account that the validation was performed on sites other than those used for algorithm development. Although the robustness of the algorithms may benefit from additional testing in more extreme situations (e.g., in other atmospheric conditions, complex topography, other crops), the developed algorithms find their strength in their simplicity. In principle it can be run continuously in near-real time over large agricultural areas without having to rely on auxiliary data. This simplicity constitutes an important advantage over radiative transfer (RT) models. RT model inversion typically needs information about the crop architectural characteristics for the generation of matching crop- and phenology-specific synthetic spectra, which is not always directly available [27,69]. Given that calibration occurred across a broad range of crop types, obtained empirical relationships are expected to be sufficiently robust for precise LAI and chlorophyll estimations. Yet, one can always strive for more powerful retrieval algorithms. For instance it would be interesting to apply and evaluate advanced non-parametric statistical models to S2 data. Over the past decades many sophisticated regression methods have been proposed; successful ones a.o. include: stepwise multiple linear regression, principal component regression, partial least square regression [41,64,70]. Furthermore, recent advances in machine learning techniques such as neural networks, support vector regression and particularly Gaussian processes regression are also very promising [55,71–73], albeit it should not be forgotten that these non-parametric approaches are equally bound to input data to train the models. Given all the above, in view of delivering improved S2 products for environmental and agriculture monitoring applications further research is planned in the directions of: (*i*) validation of the proposed algorithms along a broader range of crops and environments, (*ii*) evaluation of more advanced empirical or statistical canopy parameter retrieval models.

## 5. Conclusions

ESA’s upcoming satellite Sentinel-2 (S2) aims to replace and improve the old generation of high resolution satellite sensors Landsat and SPOT, but with improved spectral capabilities. Of specific interest for remote sensing applications for agriculture monitoring are two new bands in the red edge (B5 at 705 nm and B6 at 740 nm). In order to assess the full potential of these new bands, two empirical spectral methods that derive LAI and chlorophyll content from satellite observations have been evaluated given simulated S2 data.

First, a generic normalized difference index (NDI) was applied to estimate green LAI over agricultural sites. This optimized NDVI-like index was calculated from spectral bands centering around 665 and 705 nm, which approach the S2 B4 and B5 bands. It was demonstrated that the relationship between this index and green LAI can be approximated by a linear regression for a green LAI range that spans between 0 and 6. Additionally, the LAI- NDI relationship has been applied to airborne hyperspectral data acquired during ESA’s AgriSAR campaign. From CASI-based simulated S2 data a green LAI map has been produced and was cross-validated with *in situ* measurements of different crops with a RMSE of 0.6.

Second, the recently introduced hyperspectral index NAOC was evaluated on its capability to assess canopy level chlorophyll from airborne data of the CEFLES2 campaign, with satisfactory results. AHS airborne imagery was used and the index was calibrated with *in situ* measurements of different crops. A canopy chlorophyll map was produced based on NAOC values. At the same time, AHS data was spectrally resampled to the coarser S2 band settings and a NAOC was recalculated. Results were in close agreement with those calculated from the full spectrum AHS data. Finally, the impact of excluding the new S2 red-edge bands (B5 and B6) on the retrieval of crop chlorophyll was studied. It was found that without these bands NAOC loses its strength in accurately estimating canopy chlorophyll.

Both NDI and NAOC open opportunities to be implemented into operational S2 data processing chains with the aim of delivering high level products such as green LAI and canopy chlorophyll. The methods have been successfully tested on their robustness thanks to the availability of multiple datasets acquired from different instruments and on different agricultural sites.

## Figures and Tables

**Table 1.** S2 spectral specifications and spatial resolution [56]. The bands written in bold are those that fit within the NAOC integration limits.

**Spectral band**	**B1**	**B2**	**B3**	**B4**	**B5**	**B6**	**B7**	**B8**	**B8a**	**B9**	**B10**	**B11**	**B12**
**λ center (nm)**	443	490	560	**665**	**705**	**740**	**783**	842	865	945	1375	1610	2190
**Width band Δλ (nm)**	20	65	35	**30**	**15**	**15**	**20**	115	20	20	30	90	180
**Spatial resolution (m)**	60	10	10	**10**	**20**	**20**	**20**	10	20	60	60	20	20

**Table 2.** Specifications of the campaigns. Only the data used in this work is listed.

	**SPARC**	**AgriSAR**	**CEFLES2**
**Date**	Summer 2003, 2004	April–August 2006	April, June, September 2007
**Location**	Barrax, Spain (39°3′N, 2°6′W)	Demmin, Germany (54°0′N, 13°16′E)	Landes region, France
**Aim**	preparations for proposed SPECTRA sensor	Monitoring vegetation growth, preparations for Sentinel-1 and S2.	Preparations for CarboEurope, FLEX and S2
**Landscape**	Agricultural	Agricultural	Various landscape types: agricultural, forest, urban
**Crops**	Corn, barley, sunflower, alfalfa, wheat, onions and vegetables	Corn, winter wheat, winter rape, winter barley, sugar beet	Corn, bean, kiwi, sunflower
**Field data**	LAI	LAI	Ch
**Field instruments**	LI-COR LAI-2000 plant canopy analyzer	LI-COR LAI-2000 plant canopy analyzer	SPAD-502 chlorophyll meter
**Airborne data**		CASI (288 bands in the VNIR range, *i.e*., from 370 to 1050 nm, pixel size of 1.5 m)	AHS (63 bands in the reflective part of the electromagnetic spectrum. More info at Fernández-Renau *et al.* [62])
**Spaceborne data**	CHRIS Mode 1 (62 bands, 34 m nominal spatial resolution)	CHRIS Mode 1 (62 bands, 34 m nominal spatial resolution)	
**Preprocessing**	Images geometrically and atmospherically corrected (for details see [59])	Images geometrically and atmospherically corrected (for details see [58])	Image geometrically and atmospherically corrected (for details see [62])
